# Al_5+α_Si_5+δ_N_12_, a new Nitride compound

**DOI:** 10.1038/s41598-019-52363-7

**Published:** 2019-11-04

**Authors:** R. Dagher, L. Lymperakis, V. Delaye, L. Largeau, A. Michon, J. Brault, P. Vennéguès

**Affiliations:** 1 Université Côte d’Azur, CRHEA-CNRS, rue B. Grégory, F-06560 Valbonne, France; 2 Max-Planck-Institut für Eisenforschung GmbH, Düsseldorf, Germany; 3 Université Grenoble Alpes, CEA, LETI, MINATEC Campus, F-38054 Grenoble, France; 4 C2N-CNRS/Université Paris-Sud - Université Paris-Saclay, 10 Boulevard Thomas Gobert, 91120 Palaiseau, France

**Keywords:** Materials science, Semiconductors

## Abstract

The family of III-Nitride semiconductors has been under intensive research for almost 30 years and has revolutionized lighting applications at the dawn of the 21st century. However, besides the developments and applications achieved, nitride alloys continue to fuel the quest for novel materials and applications. We report on the synthesis of a new nitride-based compound by using annealing of AlN heteroepitaxial layers under a Si-atmosphere at temperatures between 1350 °C and 1550 °C. The structure and stoichiometry of this compound are investigated by high resolution transmission electron microscopy (TEM) techniques and energy dispersive X-Ray (EDX) spectroscopy. Results are supported by density functional theory (DFT) calculations. The identified structure is a derivative of the parent wurtzite AlN crystal where the anion sublattice is fully occupied by N atoms and the cation sublattice is the stacking of 2 different planes along <0001>: The first one exhibits a ×3 periodicity along <11–20> with 1/3 of the sites being vacant. The rest of the sites in the cation sublattice are occupied by an equal number of Si and Al atoms. Assuming a semiconducting alloy, a range of stoichiometries is proposed, Al_5+α_Si_5+δ_N_12_ with α being between −2/3 and 1/4 and δ between 0 and 3/4.

## Introduction

Due to their unique properties, wide-gap group III-Nitrides (III-N) are nowadays the materials of choice for optoelectronic applications especially for light emitting diodes which revolutionized domestic lighting in recent years^1^. They also attracted more recently a lot of attention for high power and high frequency electronic applications^2^. In most cases, devices are based on heterostructures of GaN, InGaN, and AlGaN (including AlN). Silicon plays a major role for the properties of III-N. First, Si is the most efficient n-type dopant for III-N and is therefore extensively used for III-N devices. Moreover, the introduction of Si atoms during the epitaxy drastically modifies the growth and morphology of GaN layers. The so called “SiN treatment” allows changing the growth mode from 2D to 3D^3–6^. The gradual formation of GaN pyramids leads to the bending of pre-existing dislocations and eventually to their mutual interaction and annihilation^7^. The “SiN treatment” is thus one of the most efficient methods for controling the growth and reducing the threading dislocation density in GaN heteroepitaxial layers, which is mandatory for improving the physical properties of the material up to device quality. The “SiN treatment”, implemented during the metalorganic vapor phase epitaxial (MOVPE) growth of GaN, consists in exposing the growing surface for a few minutes to a silane/ammonia flux at temperatures above 1000 °C. It was supposed that this process results in the formation of a SiN_x_ micro-mask^3^. In fact, a detailed high-resolution transmission electron microscopy (HRTEM) study revealed the formation of a crystalline monolayer-thick GaSiN_3_ compound epitaxially grown on GaN^8^. The atomic structure of this GaSiN_3_ layer may be described in reference to the parent GaN wurtzite structure: the N-sublattice is preserved whereas the three cation positions in the proposed unit cell are occupied by one Si atom, one Ga atom and one vacancy, respectively. Thus, the exposure of the GaN surface to a silane/ammonia flux does not lead to the growth of an additional layer but to the replacement of surface Ga atoms by either Si atoms or vacancies. Such a SiGaN_3_ monolayer buried by one monolayer of GaN acts as a dielectric mask and prohibits further GaN growth. As long as this SiGaN_3_ layer does not fully cover the surface, the nucleation of 3D islands occurs in areas non-covered by GaSiN_3_.

A similar “SiN treatment” has already also been successfully implemented on AlN surfaces also resulting in a further 3D growth of GaN or AlGaN^9^. Besides the importance of this process for devices based on AlGaN layers, such as UV LEDs, where the reduction of the dislocation density is crucial, one important and more fundamental question is the nature of this AlSiN layer.

The purpose of this work is to try to answer several questions which arise from the above-presented results. The first one is related to the possibility of obtaining a SiGaN crystalline layer thicker than one monolayer, while previous results have shown that a long exposure of a GaN surface to a silane/ammonia flux leads to the growth of an amorphous SiN layer^10^. On the other hand, it was reported that the *in-situ* passivation of AlGaN/GaN high electron mobility transistors leads to the growth of a crystalline Si_3_N_4_ layer^11,12^. The second question is about the possible existence of a SiAlN crystalline layer, and its exact structure.

To tackle these questions, we have studied the effect of the exposure of AlN surfaces to a silane flux at temperatures above 1300 °C. In fact, it has been shown that, at these temperatures, atom mobility is sufficiently high to promote a significant improvement of the crystalline quality of AlN layers^13,14^. This mobility may enhance the Al/Si atomic exchanges. The studied samples were (0001)-oriented AlN films grown by MOVPE or molecular beam epitaxy (MBE) on sapphire substrates and then annealed in a hot wall chemical vapor deposition (CVD) reactor and in a silicon environment. The samples were characterized by high-resolution transmission electron microscopy (HRTEM), high-resolution high-angular annular dark-field scanning TEM (HAADF-STEM), energy dispersive X-ray (EDX) spectroscopy as well as with X-ray photoelectron spectroscopy (XPS) and grazing incidence X-ray diffraction (GIXD). Density functional theory calculations were employed to investigate the atomic structure and the electronic properties of the AlSiN alloys.

## Results

The surface chemistry of the samples before and after annealing is systematically studied by X-ray photoelectron spectroscopy (XPS). First, a characteristic Si_2p_ peak appears whatever the temperature and duration of the annealing (see Fig. 1 in supplementary information (SI)) demonstrating a Si-surface enrichment. Moreover, it should be noted that the ratio of the Al_2p_ peaks after and before annealing is always below 1 indicating a decrease in the Al signal and hence suggesting that Si atoms have substituted Al ones to form an AlSiN layer.

**Figure 1:**
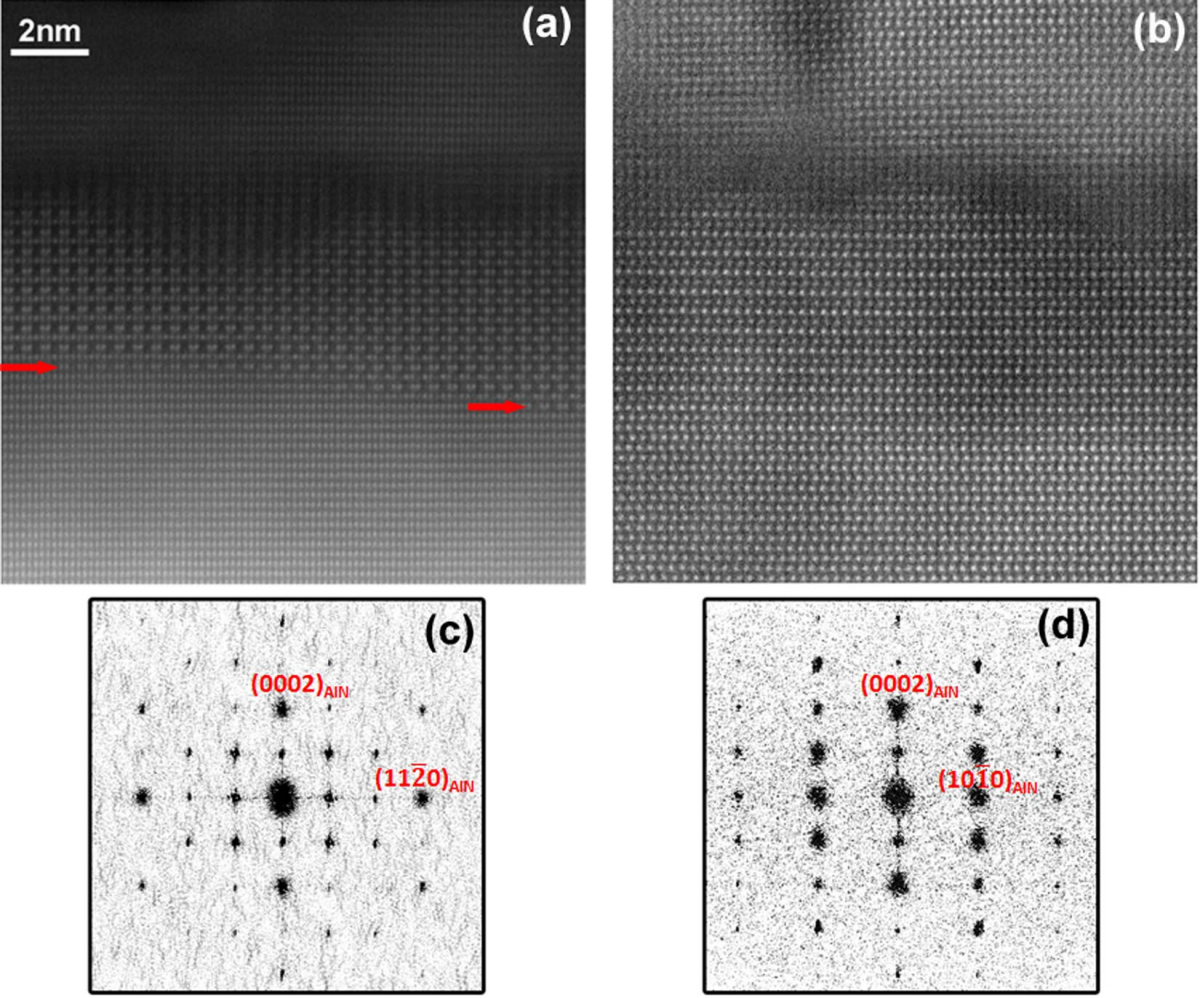
(**a**,**b**) HAADF images of the Si-rich layer and (**c**,**d**) their relative Fourier transforms: (**a**,**c**) along <10-10>_AlN_ zone axis; (**b**,**d**) along <11-20>_AlN_ zone axis. The red arrows in (**a**) indicate the first planes showing the characteristic contrast of AlSiN.

Several of these samples have been studied by cross-section TEM to determine the nature of the Si-rich surface layer. One sample has been specifically designed for TEM study and is extensively studied in the following: an MOVPE-AlN on sapphire layer has been annealed for 5 minutes at 1550 °C and then transferred to an MBE reactor. A 280 nanometer-thick AlN layer has then been overgrown to protect the Si-rich layer during TEM sample preparation. Figure 1 shows cross-section HAADF images of the Si-rich layer in this sample along two perpendicular AlN zone axes with their respective Fourier transform (FT). A characteristic contrast, which is present in all annealed AlN layers (see Fig. 2 in SI) is observed along the <10–10>_AlN_ zone axis whereas no clear difference of contrast with AlN exists along the <11–20>_AlN_ zone axis. The thickness of the layer with the aforementioned characteristic contrast varies from 4 to 6 nm along the sample. In fact, the bottom interface between this contrasted layer and AlN lies at different depths from the surface (see red arrows in Fig. 1a). The top part of this layer with a thickness of approximately 2 nm has a lower crystallinity and a darker contrast. The FT of the <10–10> HAADF image reveals a triple periodicity in the film’s plane along the <11–20> direction and a double periodicity along the <0001> direction. On the other hand, there is no additional periodicity along the <10–10> direction as shown by the FT of the <11–20> HAADF image. This in-plane triple periodicity is also observed in plan-view selected area electron diffraction (see Fig. 2 in SI). TEM describes micrometer large areas at the maximum. In order to study the structure of the AlSiN layer at a larger scale, we have performed non-coplanar GIXD which confirms the in-plane triple periodicity (see Fig. 3 in SI).

**Figure 2:**
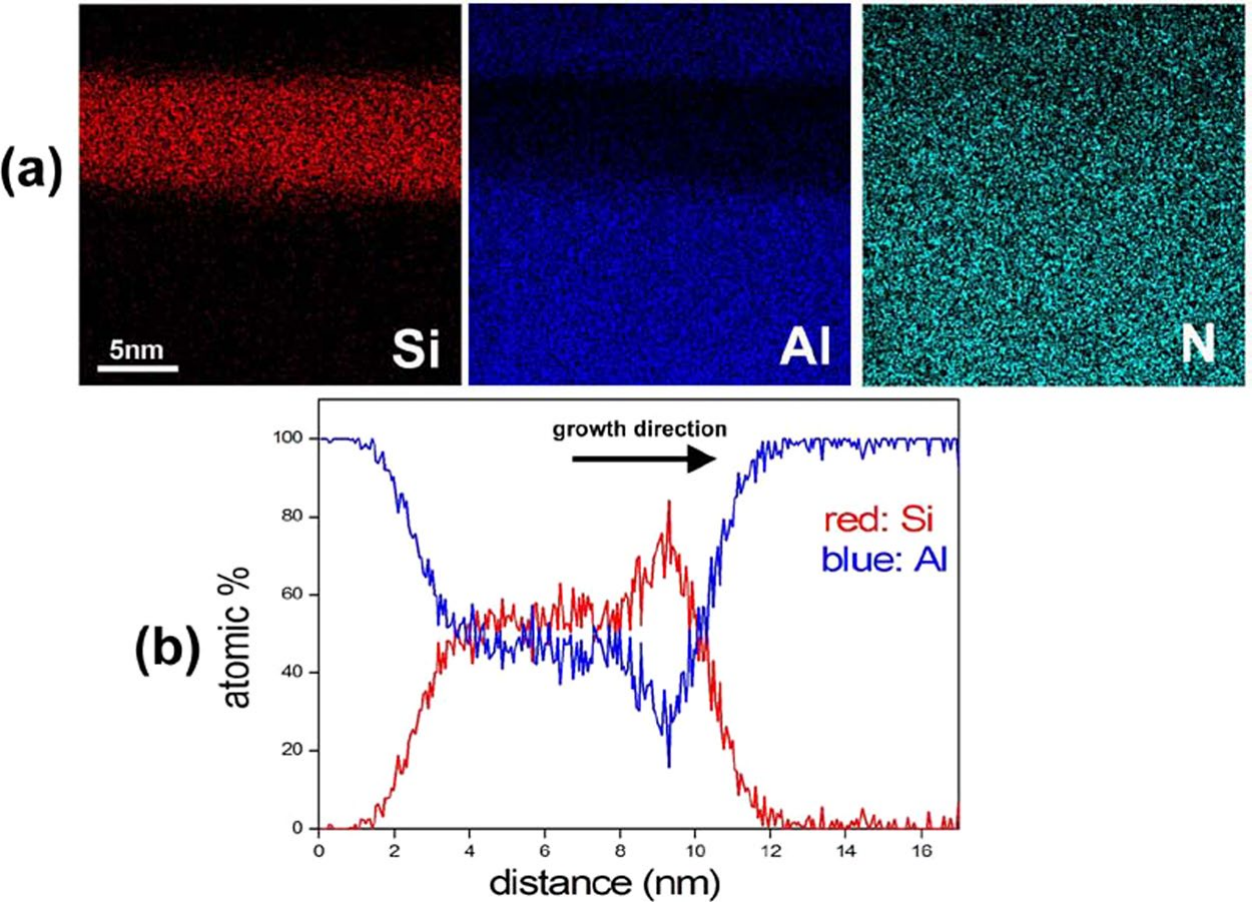
(**a**) Si, Al and N EDX maps of the AlSiN layer. (**b**) Profile of the relative Al and Si concentration across an AlSiN layer.

In order to identify the chemical composition of the AlSiN layer EDX maps have been measured. As can be seen in Fig. 2a there is an anti-correlation between Si and Al concentrations while the N concentration is nearly constant in the AlN substrate as well as in the AlSiN layer. Figure 2b is a quantitative profile across the epilayers showing the relative Al and Si concentrations. Coming from the substrate side, there is a gradual increase of the Si content up to about 50%. The position of the onset of the characteristic contrast in HAADF coincides with position in EDX profiles where the Si-concentration reaches 50%. In the top part of the layer the Si-concentration increases above 50%. However, as has already been mentioned, this region is suffering from lower crystallinity. Furthermore, a significant concentration of oxygen is also detected in this region (not shown). Nonetheless, no oxygen is detected in the bottom part of the AlSiN layer. The presence of oxygen in the upper part can be attributed to the exposure of the sample surface to air during the transfer from the CVD to the MBE reactors. In the following, we focus on the high crystallinity and oxygen free bottom part of the AlSiN layer which has a thickness of ≈4 nm. From the EDX profiles shown in Fig. 2d, the mean Al and Si contents are estimated to correspond to 47 ± 4% and 53 ± 4% of the occupied cation sublattice sites, respectively. The error bars correspond to the standard deviation from the mean values of the concentrations in the bottom 4 nm of the Si-rich layer.

**Figure 3:**
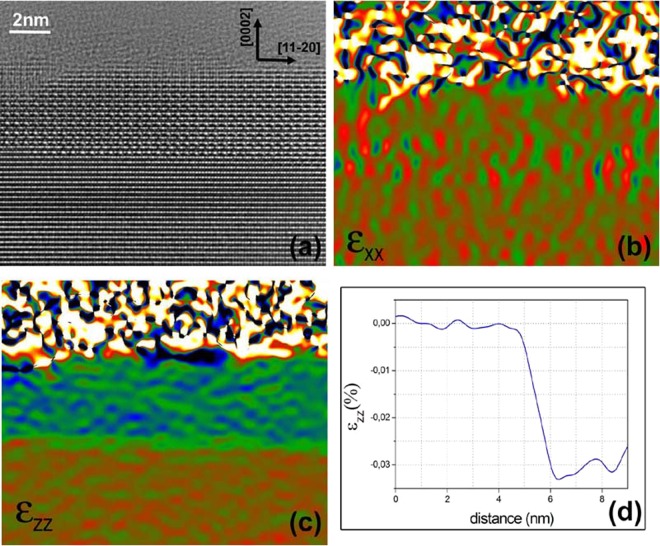
(**a)** High resolution TEM image along the <10-10>_AlN_ zone axis. (**b**) In-plane GPA strain map extracted from (**a**). (**c**) Out-of-plane GPA strain map extracted from (**a**). (**d**) Intensities in (**c**) summed up along the horizontal direction.

Figure 3a is a high resolution TEM image along the <10-10>_AlN_ zone axis. The top part of the layer has been removed during the TEM sample preparation. Figure 3b,c are strain maps of Fig. 3a obtained using the geometrical phase analysis (GPA)^15^ method for {11–20} (ε_xx_) and (0002) (ε_zz_) planes respectively. Figure 3d shows the intensities in Fig. 3c summed parallel to the interface. There is no strain for the <11–20> planes indicating that the AlSiN epilayers are fully coherent with the AlN substrate. On the other hand, the out-of plane c-lattice parameter is nearly 3% smaller than that of AlN. These observations may have 2 origins. The lattice parameters of the relaxed AlSiN structure may be equal to those of AlN in the interface plane and 3% smaller perpendicularly or AlSiN may be elastically strained to be pseudomorphic with the AlN substrate.

**Figure 4:**
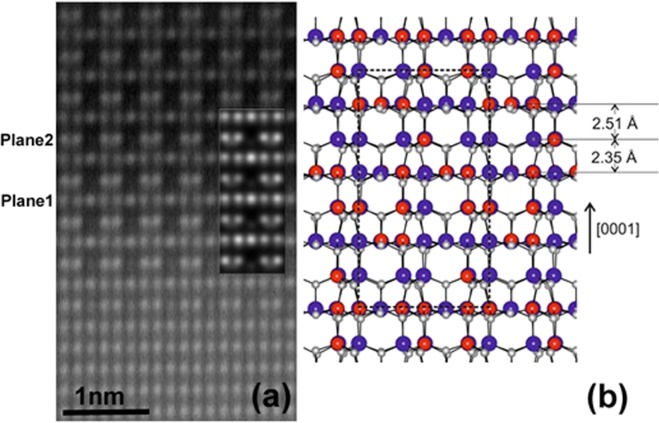
(**a**) Wiener-filtered HAADF image of the bottom part of the AlSiN layer along the <10-10>_AlN_ zone axis. The insert shows a simulated image using the model presented in (**b**). (**b**) Schematic representation of the relaxed Si_5_Al_5_N_12_ structure in the <1-100> projection. The dashed rectangle indicates the unit cell. Large red and blue spheres denote Si and Al atoms, small gray spheres the N atoms. The average interplane distances of the successive layers are also indicated.

Figure 4a shows a characteristic high magnification Wiener-filtered HAADF image of the Si-rich layer along the <10-10>zone axis. The structure may be described as the stacking of 2 different planes, hereafter referred to as plane 1 and plane 2, along the vertical direction corresponding to the observed double periodicity. Plane 1 shows a homogeneous contrast of the atomic columns with intensities similar to those observed in the AlN substrate. However, a triple in-plane periodicity is clearly observed in plane 2. Every third column in this plane appears dark with almost no intensity while the intensity of the other two columns are similar to those of AlN and therefore to those in plane 1. With the acquisition conditions used, HAADF images show Z-contrast with atomic column intensities proportional to Z^α^, Z being the average atomic number along the column and with α being between 1.6 and 2. Since Al and Si atoms have close atomic numbers (13 and 14 respectively), it is not possible to distinguish Al-rich or Si-rich columns.

## Discussion

From the above experimental inputs, a structural model can be proposed for the AlSiN structure. First, the compact hexagonal N sublattice is assumed to be unchanged. In plane 1, all atomic columns exhibit similar contrast in HAADF images and EDX indicates nearly the same Al and Si concentrations: the same number of Al and Si atoms is then distributed in the cation sites. Plane 2 presents a triple periodicity similar to the GaSiN_3_ monolayer observed by Markurt *et al*.^8^ suggesting a comparable structure. The empty cation position corresponds to the atomic column position showing no intensity in the <10-10> HAADF image. In the case of GaSiN_3_, the other two cation positions are occupied by either a Si or a Ga atom. As has already been mentioned, it is impossible from our data to distinguish between Si- or Al- rich columns. Therefore, in the following discussion a random distribution of Si and Al atoms is assumed in the occupied sites of the cation sublattice.

A schematic representation of the structure resulting from DFT calculation is shown in Fig. 4b. In accordance with the experimentally identified dominant structural characteristics, the cation sublattice is a stacking of two alternating layers along the c-direction. Both layers contain an equal number of Si and Al atoms randomly distributed. However, 1/3 of the cation sites in plane 2 are vacant. The vacancies are distributed in an ordered $\sqrt{3}\times \sqrt{3}$ pattern and the structure stoichiometry is Si_5_Al_5_N_12_. The out of plane lattice constant is ≈2% contracted with respect to the corresponding lattice constant of AlN. This lattice contraction is in good agreement with experimental observations and the difference can be attributed to a larger concentration of the cation vacancies in the calculated structure than in the actual structure (see below). The relaxed structure has been used as input for an HAADF image calculation. The insert in Fig. 4a shows a simulated image for a specimen thickness of 5 nm. The good fit between the simulated and the experimental images allows to validate the proposed structural model.

Although the exact stoichiometry of the AlSiN structure cannot be obtained directly from the aforementioned experiments, under the assumptions that (i) the anion sublattice is fully occupied and (ii) the AlSiN structure is semiconducting, a good estimation can be made. If we assume that the actual stoichiometry is Al_x_Si_y_N_12_ then in order to achieve a semiconducting AlSiN layer the electron counting rule (ECR) should be applied, i.e. all bonding and anion dangling bond states should be occupied. The total number of valence electrons per structural unit in the Al_x_Si_y_N_12_ layer is n_e_ = 3x + 4 y + 60, where the last term is the total number of valence electrons contributed by the twelve N atoms. Moreover, the total number of electrons required to occupy all Si-N and Al-N bonding states as well as all N dangling bond states is 8 × 12 = 96 (12 N-sites and 12 cation-sites in the unit cell). Therefore, obeying ECR leads to 3x + 4 y = 36. If we assume equal number of Si and Al atoms then x = y ≈ 5.14 and in this case ≈14.3% of the cation sites should be vacant. This is somewhat smaller than the 16.6% cation vacancies required if the dark columns in plane 2 consist of vacancies only. The later could be achieved only if y = 6 and x = 4, i.e. if the Si and Al contents in the Al_x_Si_y_N_12_ structure are 50% and 33% of the cation sites, respectively which is not what is experimentally observed. A few Al and/or Si atoms are presumably present in the sites corresponding to the dark columns.

As has already been mentioned, the mean Al and Si contents are estimated to correspond to 47 ± 4% and 53 ± 4% of the occupied cation sublattice sites, respectively. Applying the ECR to these compositions we find that Al_5.26_Si_5.05_N_12_ and Al_4.34_Si_5.75_N_12_ correspond to the extremes of Al rich and Si rich stoichiometries, respectively. These structures contain ≈14% and ≈16% cation vacancies, respectively. We can therefore propose a stoichiometry Al_5+α_Si_5+δ_N_12_ with α being between ≈−2/3 and ≈1/4 and δ between ≈0 and ≈3/4.

**Figure 5:**
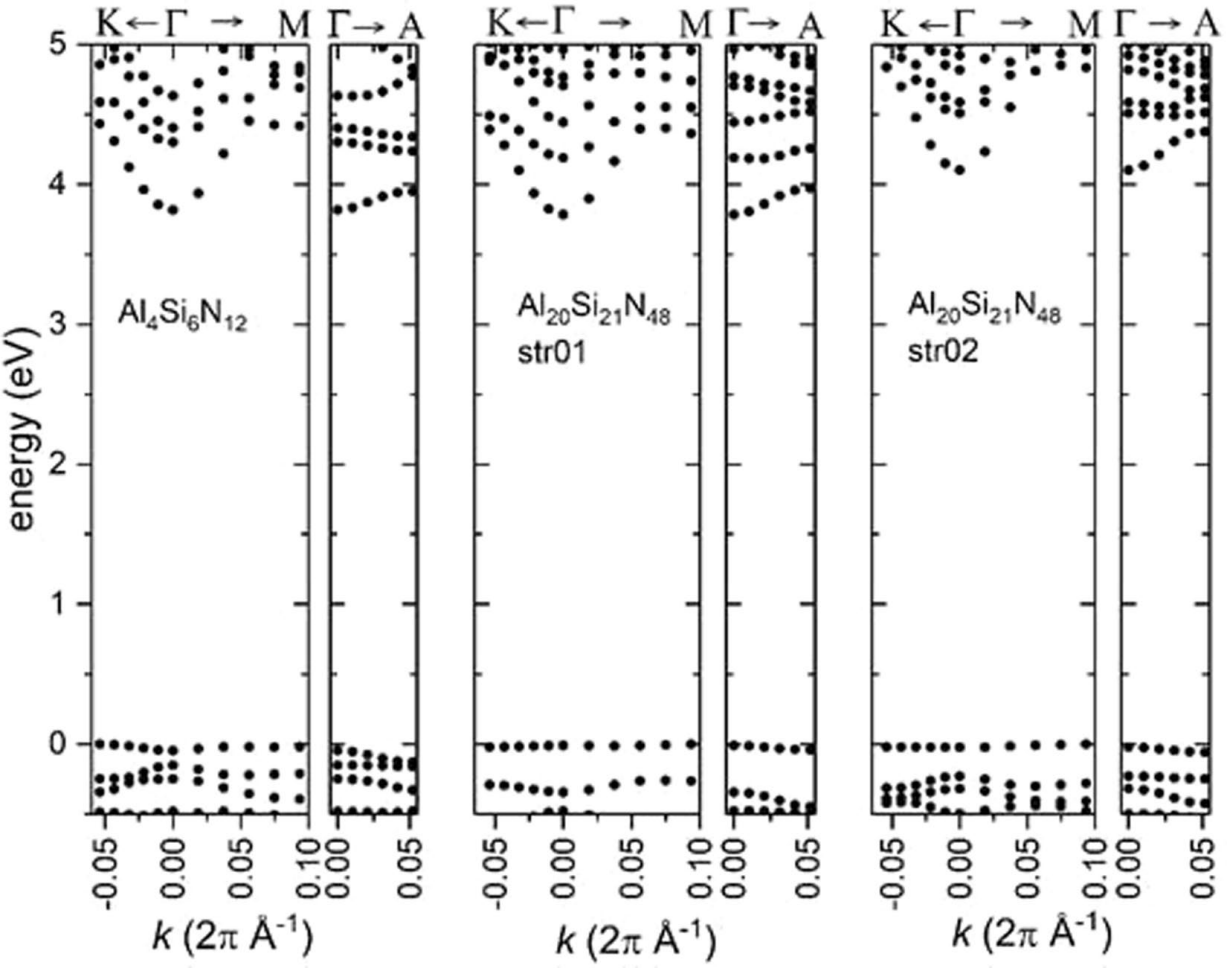
Calculated band structures of selected Al_5+α_Si_5+δ_N_12_ alloys. All structure obey ECR and have the $2\sqrt{3}\times 2\sqrt{3}\times 2$ unit cell. All plots have been shifted such that the edges of the valence bands are at 0 eV.

In order to gather insights in the electronic properties of these alloys we have calculated the bandstructures of two selected Al_5+α_Si_5+δ_N_12_ structures with α = 0 and δ = 1/4 that obey ECR. These structures differ by the distribution of Al/Si atoms. For comparison we have also calculated the bandstructure of an alloy where all dark columns in plane 2 consist of vacancies only (Al_4_Si_6_N_12_). The bandstructures are shown in Fig. 5 and as can be seen in all cases Al_5+α_Si_5*+δ*_N_12_ has a wide bandgap of the order of ≈3.8–4.4 eV. Although the calculated alloy structures do not necessarily correspond to ground state configurations and/or stoichiometries, the above results provide strong evidence that Al_5+α_Si_5+δ_N_12_ alloys are wide bandgap materials with a bandgap that most probably exceeds that of GaN. Furthermore, a common feature in all calculated bandstructures is the almost dispersionless character of the valence band maximum indicating very large values for the hole effective masses.

A few issues on the proposed structural model and stoichiometry as well as on the microstructure of the AlSiN layers have to be further addressed. Firstly, the vacant cation sites of the proposed structural model presents a triple periodicity in the film plane and a double periodicity perpendicular as compared to the wurtzite one of the AlN templates. Therefore, translational domains, such as those formed, for example, when planes 1 and 2 are shifted by 1/2 c_AlN_ in adjacent domains, cannot be excluded in the microstructure of the AlSiN layer. Secondly, we have assumed a random distribution of Al and Si on the cation sites. This assumption cannot be validated by HAADF experiments owing to the very similar Al and Si atomic masses. However, disordered or ordered distributions of Al and Si atoms and/or small changes in the exact stoichiometry may have drastic consequences on the physical properties of AlSiN and should therefore be investigated. These issues are beyond the scope of the present paper.

Nevertheless, the above mentioned preliminary results already point to potential technological applications. First of all, the formation of a non-continuous AlSiN layer may lead to a 3D overgrowth of Al(Ga)N layers and, in a similar way as for GaN, to a drastic reduction of the dislocation density. Moreover, AlSiN seems to be a new semiconductor with a gap around 4 eV. Although this is not completely clear, it might be lattice matched to AlN. Compared to Al_0.2_Ga_0.8_N that has a similar gap of 4 eV, and which has a lattice mismatch of about 1.6% with AlN, this can be advantageous for growing heterostructures with AlN barriers. Due to its possibly disordered structure in terms of Al and Si position, one can conjecture that the spontaneous polarization could be smaller than in usual nitrides. If we add a small strain when grown on AlN, this could lead to a total polarization field much smaller than in GaN/AlN or AlGaN/AlN quantum wells, which could be beneficial for light emitters or normally off transistors. The flat valence band suggests heavy hole masses and a large joint density of states, which means large absorption coefficients. This would not be favorable for lasers but could be interesting for detectors. Finally, carrier and in particular hole localization on a nanometer scale might be interesting to study in such AlSiN layers if the Al and Si distribution is not homogenous. Such a topic would be particularly interesting in the frame of UV emitters.

## Conclusions

In conclusion, we have shown that the high temperature annealing of AlN epitaxial layers under a Si atmosphere makes it possible to synthesize a new Nitride compound. Based on HRTEM, HAADF-STEM, EDX measurements and DFT calculations a structural model and a stoichiometry (Al_5+α_Si_5+δ_N_12_) are proposed. Preliminary calculations indicate that this new compound is a wide gap semiconductor with a gap larger than that of GaN. A detailed investigation of the properties of this new material is appealing both from a fundamental point of view as well as to assess potential applications.

## Methods

### Synthesis

(0001)-oriented AlN heteroepitaxial films grown on sapphire either by MBE^16^ or MOVPE^17^ are annealed in a hot wall chemical vapor deposition (CVD) reactor in a silicon environment. The presented results do not depend on the growth technique of the AlN template layers. Two different processes are used, process 1 and process 2. Before process 1, the clean CVD reactor is exposed to a silane flux for 5 minutes at 1350 °C and 200 mbar in a H_2_ atmosphere. Then, process 1 consists in a simple annealing under N_2_ flux at 1550 °C and 800 mbar for a few minutes without silane. Process 2 is similar to process 1, except that in addition to the pre-exposure of the reactor to silane, the annealing is performed under a silane flux varying between 0.5 and 5 sccm. In this study, both the duration and the temperature of the annealing have been varied, as can be seen in Fig. 1 of supplementary information (SI).

### TEM investigations

TEM specimens are prepared using a conventional technique involving mechanical thinning followed by ion milling using Ar at 0.5–5 keV. TITAN THEMIS microscopes operated at 200 kV and fitted either with probe or with objective corrector are used enabling spatial resolution below 0.1 nanometer. The probe corrected one is also fitted with a high sensitivity energy dispersive X-Ray (EDX) spectroscopy system. The HAADF images have been acquired using a half-beam convergence of 20 milliradians, a camera length of 110 millimeters and a detector half-angle width extending from 65 to 200 milliradians. HAADF image simulations have been performed with the JEMS software^18^ using the multislice method and the frozen phonon approximation with the experimental parameters indicated above. The AlN on sapphire samples are very insulating and therefore subject to charging effects during TEM observations. To overcome this problem, short exposure images have been acquired. The presented high resolution high angle annular dark field (HAADF) scanning TEM images are in fact the sum of 10 single images.

### Ab-initio investigations of Al_5+α_Si_5+δ_N_12_

Density functional theory (DFT) calculations within the local density approximation (LDA) for the exchange and correlation and the projector augmented-wave (PAW) method have been employed in order to calculate the relaxed atomic geometry of the experimentally suggested structure of the AlSiN layers^19,20^. The AlSiN layers have been modeled using supercells consisting of 8 N-cation ML along <0001> with a $2\sqrt{3}\times 2\sqrt{3}$ periodicity in the basal plane. The plane-wave energy cutoff was 450 eV and an equivalent of a 6 × 6 × 1 Monkhorst-Pack k-point mesh for the unit cell was used to sample the Brillouin zone (BZ). All the atoms in the supercells have been allowed to relax until the forces are smaller than 1 meV/Å. Furthermore, the AlSiN layers have been assumed to be biaxially strained to AlN, i.e. the in-plane lattice constant is fixed to the lattice constant of AlN and the supercells were allowed to relax the strain along the <0001> direction. The electronic structure of selected $2\sqrt{3}\times 2\sqrt{3}\times 2$ unit cells of Al_5+α_Si_5+δ_N_12_ structures that obey the electron counting rule been computed with the Heyd, Scuseria, and Ernzerhof hybrid functional (HSE) with 25% exact exchange^21^ and a plane-wave energy cutoff of 400 eV. The calculated bulk bandgap for AlN is 5.88 eV.

## Supplementary information

Supplementary information
