# Supplementary material for: Al5+αSi5+δN12, a new Nitride compound
Source: Sci Rep. 2019 Nov 4;9:15907. doi: 10.1038/s41598-019-52363-7 (PMC6828660; doi:10.1038/s41598-019-52363-7)
Supplement: Supplementary file 1 — Supplementary information [file 41598_2019_52363_MOESM1_ESM.pdf]

$Al_{5+\alpha}Si_{5+\delta}N_{12}$, a new Nitride compound

Supplementary information

R. Dagher[1,3], L. Lymperakis[2], V. Delaye[3], L. Largeau[4], A. Michon[1], J. Brault[1] and P. Vennéguès[1]

[1] Université Côte d'Azur, CRHEA-CNRS, rue B. Grégory, F-06560 Valbonne, France

[2] Max-Planck-Institut für Eisenforschung GmbH, Düsseldorf, Germany

[3] Université Grenoble Alpes, CEA, LETI, MINATEC Campus, F-38054 Grenoble, France

[4] C2N-CNRS / Université Paris-Sud - Université Paris-Saclay, 10 Boulevard Thomas Gobert, 91120 Palaiseau, France

X Ray Photoelectron spectroscopy

The surface chemistry of AlN layers has been investigated by ex-situ X-ray Photoelectron spectroscopy as a function of the annealing conditions. Figure 1 gives examples of Si_{2p} and Al_{2p} XPS spectra and the ratio of Si_{2p} (Al_{2p}) intensities after and before annealing. The ratio is greater than one for Si (Si-enrichment of the surface) and lower than 1 for Al (Al-impoverishment of the surface). The optimum conditions for surface Si enrichment (highest Si_{2p} peak intensity) are obtained in process 2 with 5 sccm of SiH_4 and at 1550°C for a duration between 5 and 15 minutes.

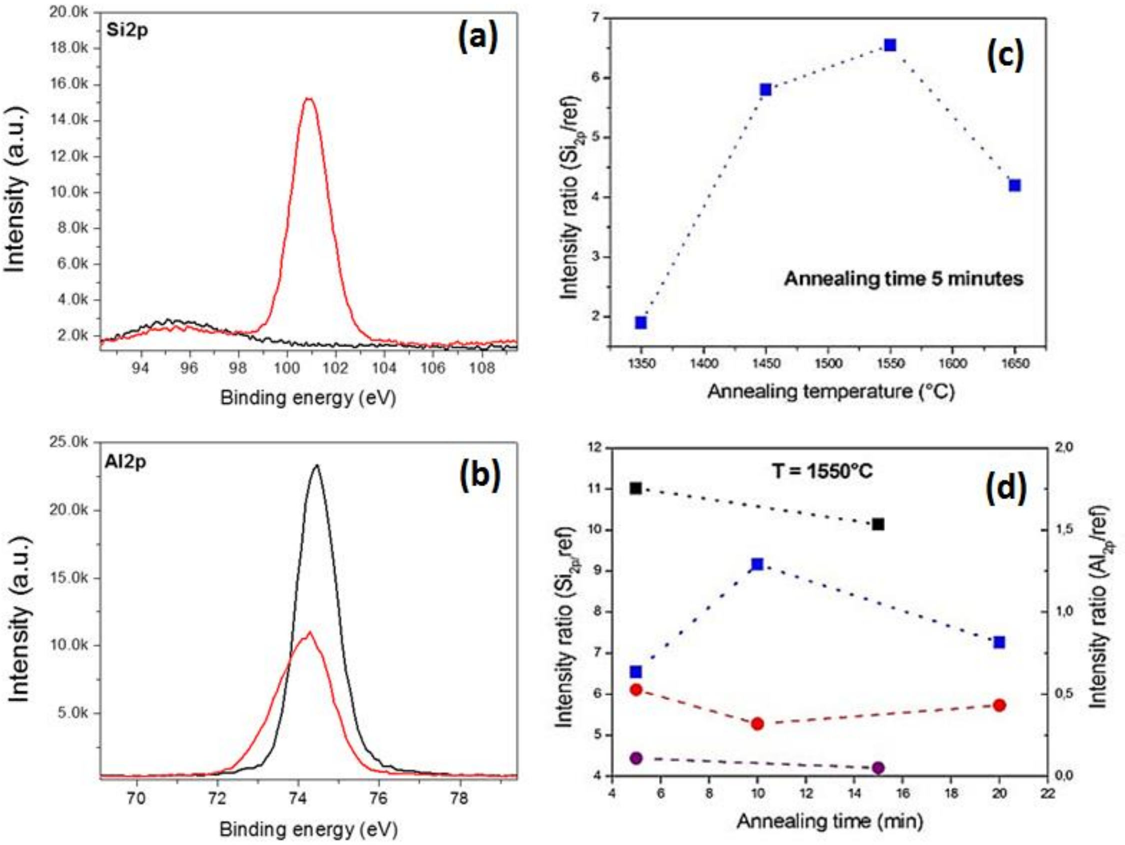

Figure 1 SI.

(a) and (b): Si_{2p} and Al_{2p} XPS spectra before (black) and after (red) an annealing at 1550°C for 5 minutes

(c) ratio between the Si_{2p} peaks after and before (noted as "ref") annealing as a function of the temperature for a 5 minute annealing

(d) ratio between the Si_{2p} (resp. Al_{2p}) peaks after and before (noted as "ref") annealing as a function of the time for an annealing at 1550°C. In (d), the square symbols correspond to Si_{2p} ratio (left scale) for process 1 (blue) and 2 (black) whereas round symbols correspond to Al_{2p} ratio for process 1 (purple) and 2 (red).

Surface AlSiN layer

The paper is focused on the study of AlSiN layer embedded in AlN. AlSiN layers are also clearly detected by TEM even without an AlN overgrowth. Figure 2 SI shows the presence of such an AlSiN layer for an annealing of a MBE-grown AlN layer for 5 minutes at 1550°C with process 1. This sample has also been studied by plan-view TEM. The plan-view selected area electron diffraction pattern shows the triple periodicity of AlSiN along the <11-20> directions.

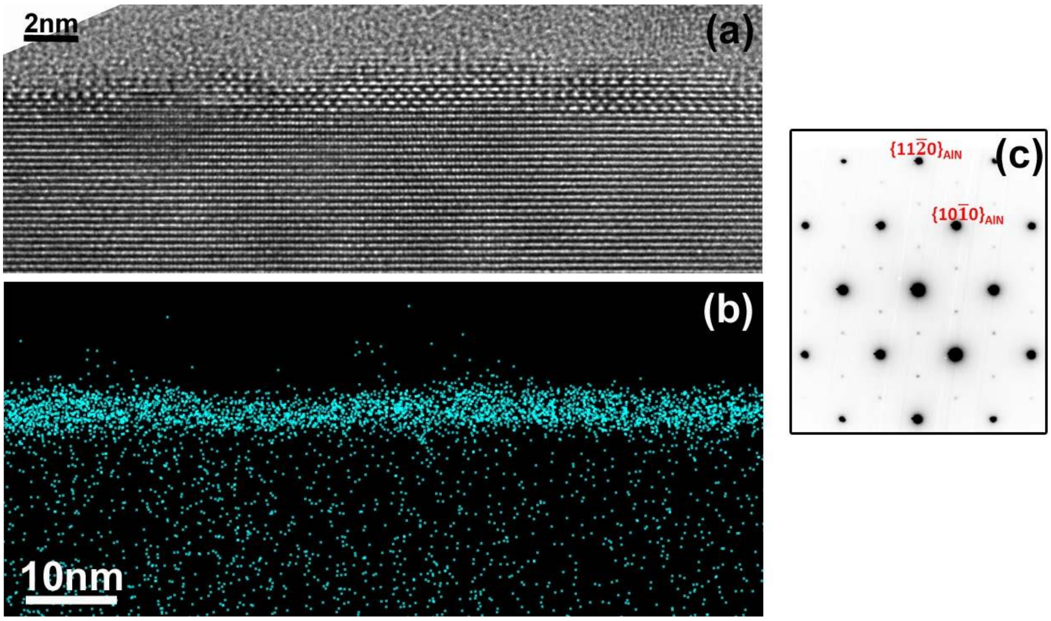

Figure 2 SI.

(a) High resolution TEM image of an MBE-AlN layer annealed at 1550°C with process1
(b) Si EDX map
(c) Plan-view selected area electron diffraction pattern

To study the structure of such a surface layer at large scale non-coplanar GIXD has been performed using a Rigaku Smartlab 5-axes diffractometer equipped with a rotating anode allowing diffraction of planes perpendicular to the surface. A 2-bounds Ge (400) monochromator has been used for selecting Cu-Kα1 diffraction peaks. The instrumental resolution has been fixed by collimators at 0.5°. Figure 3 SI (a) shows a 2ThetaChi/Phi scan collected along the in-plane [11-20] AlN azimuthal direction. The AlN diffraction peak appears at 2ThetaChi=59.52° which corresponds to a d value of 0.1552nm. An additional peak is visible at 2ThetaChi=19.08°. This additional peak corresponds to a d value of 0.4647 nm which is exactly 3 times the d value of AlN. This additional peak corresponds to the triple crystalline periodicity observed by (S)TEM which is then present over large surfaces. We have also performed Phi scans (shown in figure 3 SI (b)) with the detector fixed at the 2ThetaChi value corresponding to this in-plane triple periodicity peak. We observed a 6-fold symmetry corresponding to the hexagonal structure of AlN. Interestingly, no additional peak has been

observed along the in-plane AlN [10-10] azimuthal direction indicating that the in-plane periodicity takes place only along the in-plane <11-20> directions.

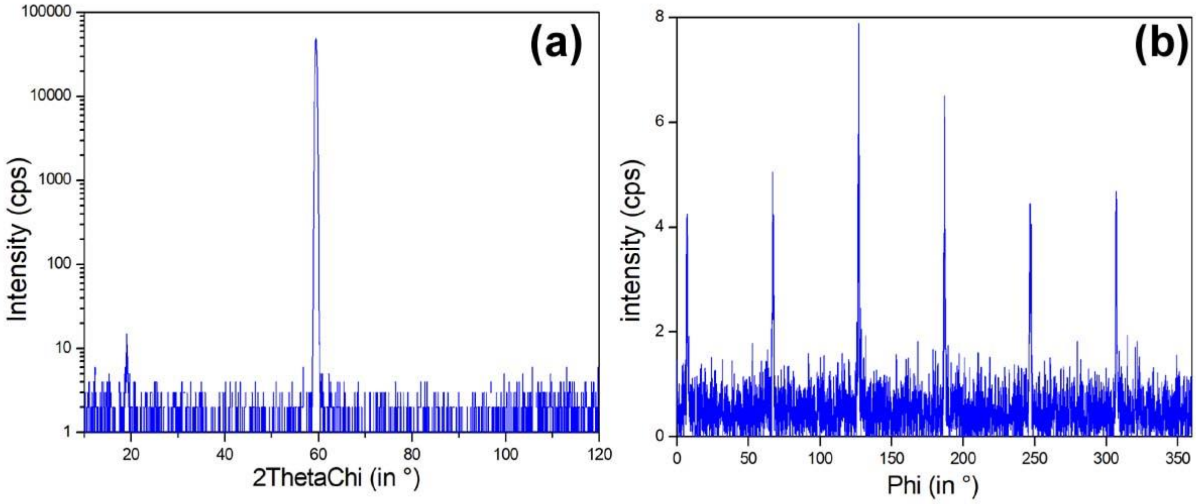

FIGURE 3 SI.

GIXD spectra of an AlN sample annealed at 1500°C for 5 minutes

a) 2ThetaChi/Phi scan

b) Phi scan
